# Crystal structure and computational study of 1-hy­droxy-3,6,7-trimeth­oxy-2,8-bis­(3-methyl­but-2-en-1-yl)-9*H*-xanthen-9-one (fuscaxanthone C)

**DOI:** 10.1107/S2056989026004792

**Published:** 2026-05-15

**Authors:** Wei Chung Sim, Huey Chong Kwong, Gwendoline Cheng Lian Ee, Mohamed Ibrahim Mohamed Tahir

**Affiliations:** a Malaysian Agricultural Research & Development Institute KKIP 88460 Kota Kinabalu Sabah Malaysia; b Department of Chemistry, Faculty of Science, Universiti Putra Malaysia, 43400 UPM Serdang, Selangor Darul Ehsan, Malaysia; University of Aberdeen, United Kingdom

**Keywords:** crystal structure, natural product, xanthone, C—H⋯π inter­action, ketone-O⋯ π inter­action, Hirshfeld surface analysis

## Abstract

The title xanthone derivative, C_26_H_30_O_6_, extracted from the bark of *Cratoxylum glaucum*, adopts an almost planar fused ring system with a maximum deviation of 0.078 (1) Å. In the crystal, mol­ecules are linked into inversion dimers by pairwise C—H⋯O inter­actions. These dimers are further linked into chains by C—H⋯π and C—O⋯π inter­actions. Computational chemistry revealed that the overall stabilization energy of the packing is dominated by dispersion energy. The conformations of related xanthones are also surveyed.

## Abstract 2

The title compound consists of a xanthone fused ring system with hy­droxy, meth­oxy and 3-methyl­butenyl substituents. In the crystal, the mol­ecules are linked by C—H⋯O, C—H⋯π and C—O⋯π inter­actions.

## 1. Chemical context

Fuscaxanthone C, C_26_H_20_O_6_, is a yellow compound initially synthesised and reported as di­methyl­mangostin (Yates & Stout, 1958 ▸). It was later renamed as fuscaxanthone C, after its isolation as a natural product from *Garcinia fusca* (Ito *et al.*, 2003 ▸). This compound has also been discovered to be present in the bark of *Cratoxylum glaucum* and *Cratoxylum arborescens* (Sim *et al.*, 2011 ▸) and *Calophyllum benjaminum* (Sahimi *et al.*, 2015 ▸), three plants from Sarawak, Malaysia, as well as *Garcinia cowa* (Siridechakorn *et al.*, 2012 ▸) from Nong Khai, Thailand. In this study, fuscaxanthone C was extracted from the bark of *Cratoxylum glaucum*, collected from Sarawak, Malaysia*. C. glaucum*, locally known as ‘ketemau’ or ‘geronggang timau’ (Iban), is one of the six *Cratoxylum* species indigenous to Southeast Asia (Wong, 1995 ▸, Boonnak *et al.*, 2006 ▸). All six species can be found in Borneo. The name *Cratoxylum* is derived from two Greek words, *kratos* meaning strong and *xylon* meaning wood. The hard and durable wood of *Cratoxylum* is generally classified in the timber industry as derum (heavy timber) and geronggang (light timber). *Cratoxylum* stem bark usually exudes a yellow resinous sap which turns black when dry and has been applied in traditional medicine by the local people of Malaysia (Wong 1995 ▸; Bennett *et al.*, 1993 ▸). The bark, roots and leaves of *Cratoxylum* species have also been reported to be used in the treatment of itches, ulcers, fevers, cough, diarrhoea, and abdominal complaints (Nguyen & Harrison, 1999 ▸). *Cratoxylum* species have also demonstrated anti­oxidant (Sim *et al.*, 2011 ▸), anti­malarial (Laphookhieo *et al.*, 2009 ▸), anti­bacterial (Boonsri *et al.*, 2006 ▸), cytotoxic (Pattanaprateeb *et al.*, 2005 ▸), anti-HIV-1 (Reutrakul *et al.*, 2006 ▸) and anti­diabetic (Lv *et al.*, 2019 ▸) properties. In a continuation of our studies on natural products and their derivatives (Ee *et al.*, 2010 ▸), we report herein the crystal structure and Hirshfeld surface analysis of the title compound, C_26_H_20_O_6_ (**I**).
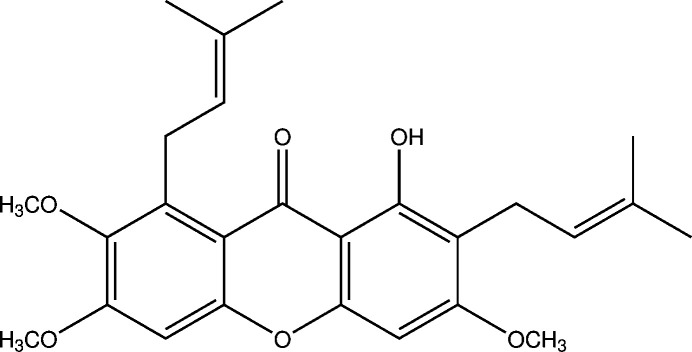


## 2. Structural commentary

**Figure 1:**
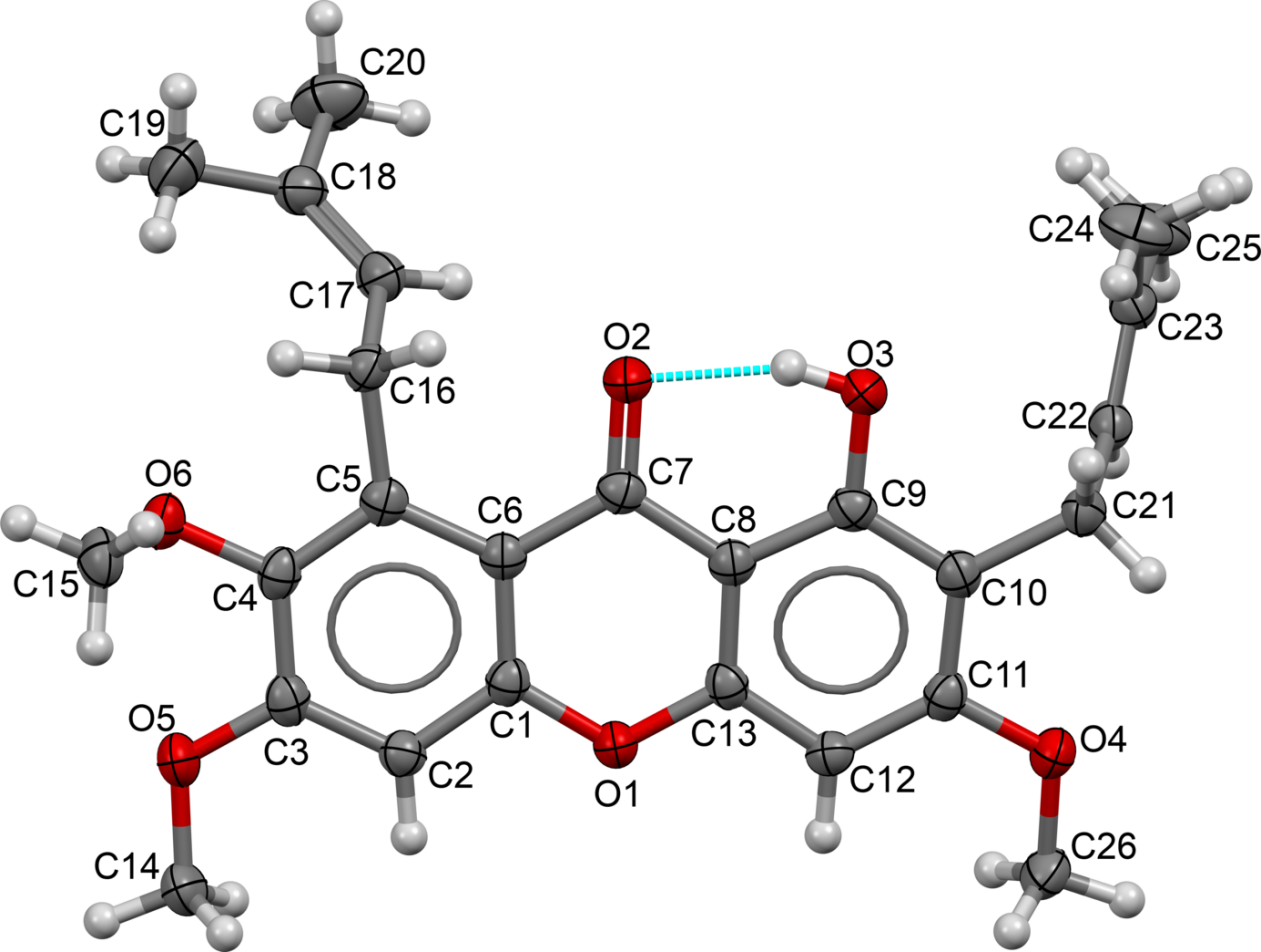
The mol­ecular structure of (**I**) with displacement ellipsoids drawn at the 50% probability level.

Compound (**I**) crystallizes in the centrosymmetric triclinic space group *P*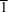 and its asymmetric unit consists of a single unique mol­ecule (Fig. 1 ▸). The C1–C13/O1 xanthone fused ring system is approximately planar (r.m.s. deviation = 0.045 Å) with a maximum deviation of 0.078 (1) Å at atom C11. The dihedral angle between the C1–C6 and C8–C13 phenyl rings is 4.63 (3)°. The C atoms of the meth­oxy substituents attached to C3 and C11 are almost coplanar with their attached rings, as indicated by the C2—C3—O5—C14 and C12—C11—O4—C26 torsion angles of −3.2 (2) and 4.87 (18)°, respectively. Conversely, the C atom of the meth­oxy substituent attached to C4 is substanti­ally displaced from the ring with a C3—C4—O6—C15 torsion angle of 75.47 (15)°. The C16–C20 3-methyl­but-2-enyl substituent attached to the xanthone ring system at C5 forms a C4—C5—C16—C17 torsion angle of −101.46 (14)° indicating a (–)-anti­clinal conformation. The other 3-methyl­but-2-enyl substituent attached to C10 exhibits a C9—C10—C21—C22 torsion angle of −78.58 (16)°, which indicates a (–)-synclinal conformation. The *sp*^2^-hybridized character of atoms C17, C18, C22 and C23 are confirmed by the C17=C18 [1.328 (2) Å] and C22=C23 [1.330 (2) Å] bond lengths and the C16—C17—C18 [127.38 (12)°] and C21—C22—C23 [127.38 (13)°] bond angles. In the mol­ecule, the O3—H1*O*3 hy­droxy group act as an hydrogen bond donor to atom O2 of the adjacent ketone group, thus forming an intra­molecular hydrogen bond with an *S*(6) ring motif (Table 1 ▸, Fig. 1 ▸).

## 3. Supra­molecular features

**Figure 2:**
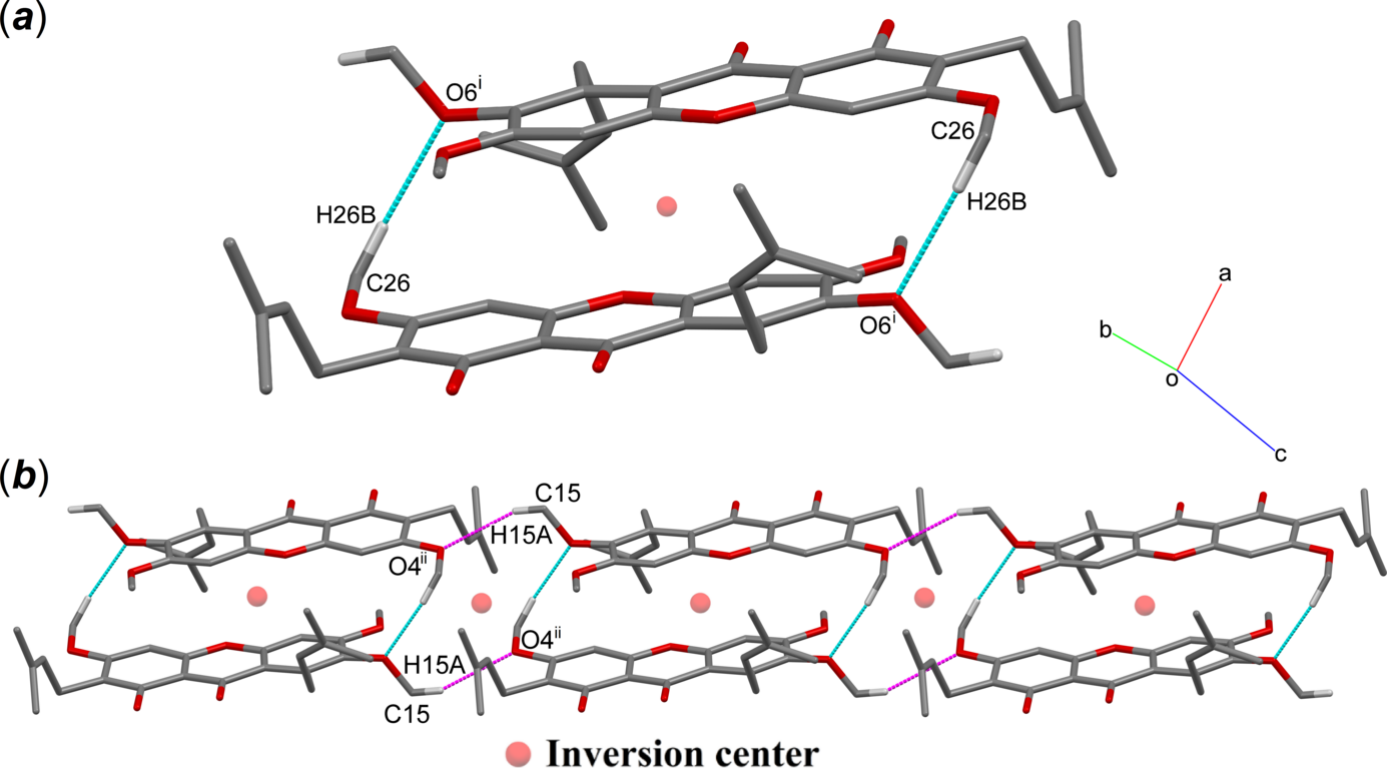
A partial packing diagram of (**I**) showing (*a*) C26—H26*B*⋯O6 and (*b*) C15—H15*A*⋯ O4 inter­actions (dotted lines). Hydrogen atoms not involved in these inter­actions are omitted for clarity.

**Figure 3:**
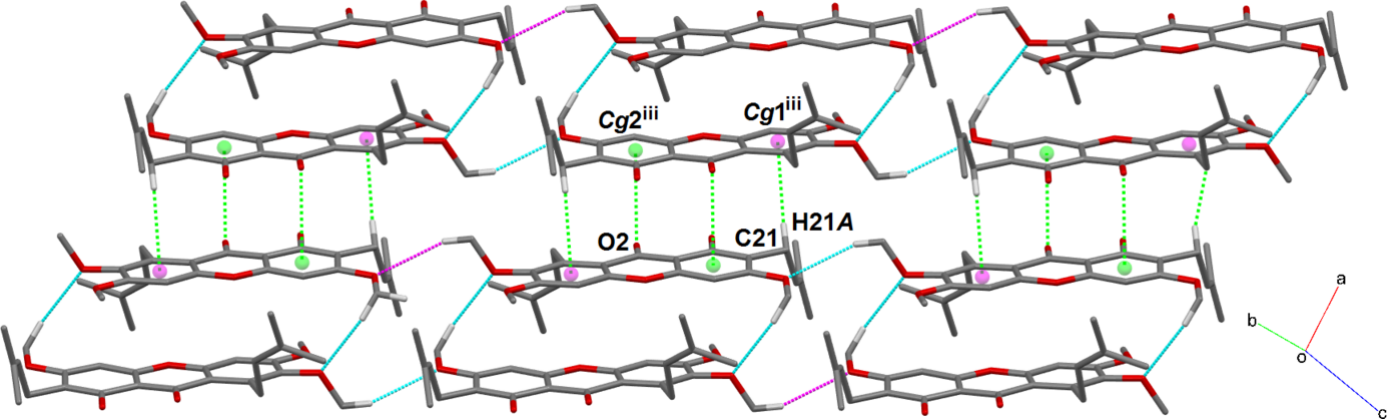
A view of a dimeric assembly in (**I**) with C21—H21*A*⋯*Cg*1 and ketone-O2⋯*Cg*2 inter­actions shown as green dotted lines. *Cg*1 and *Cg*2 are shown as magenta and green spheres, respectively.

In the crystal, the mol­ecules of (**I**) are linked into inversion dimers by weak methyl-C26—H26*B*⋯O6 (meth­oxy) inter­actions (Fig. 2 ▸*a*). These dimers are connected into chains propagating along [101] by weak C15—H15*A*⋯O4 (meth­oxy) inter­actions (Fig. 2 ▸*b*). These inter­actions form 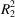
(24) and 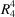
(12) ring motifs, respectively. Meanwhile, these dimeric chains are connected into a two-dimensional array lying parallel to the *b* axis via methine-C21—H21*A*⋯*Cg*1 and ketone—O2⋯*Cg*2 inter­actions (Fig. 3 ▸) (*Cg*1 and *Cg*2 are the centroids of the C1–C6 and C8–C13 benzene rings, respectively).

## 4. Hirshfeld surface analysis

**Figure 4:**
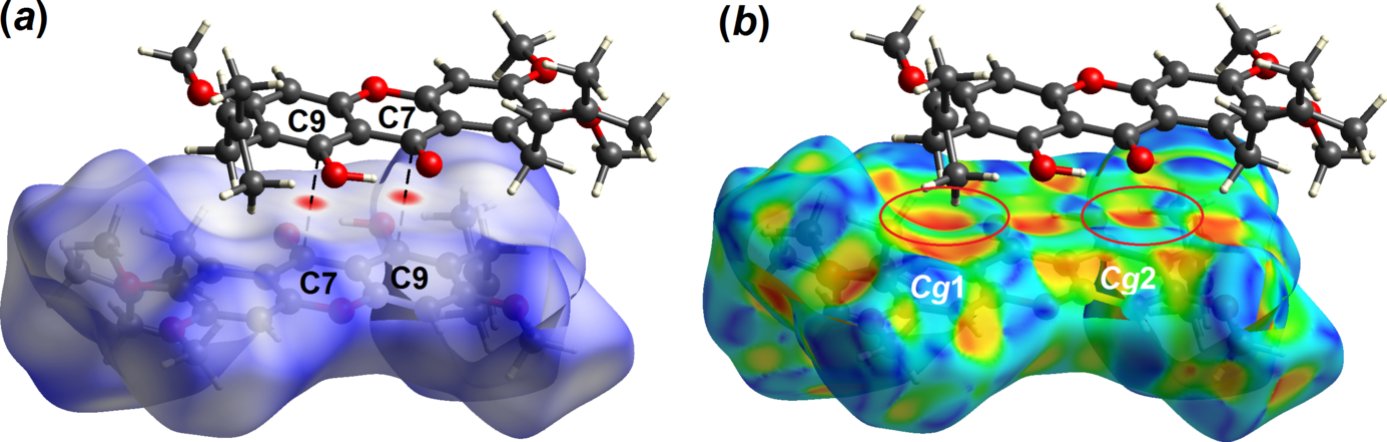
The Hirshfeld surface of (**I**) mapped over (*a*) *d*_norm_ and (*b*) shape-index showing C—H⋯π inter­actions.

**Figure 5:**
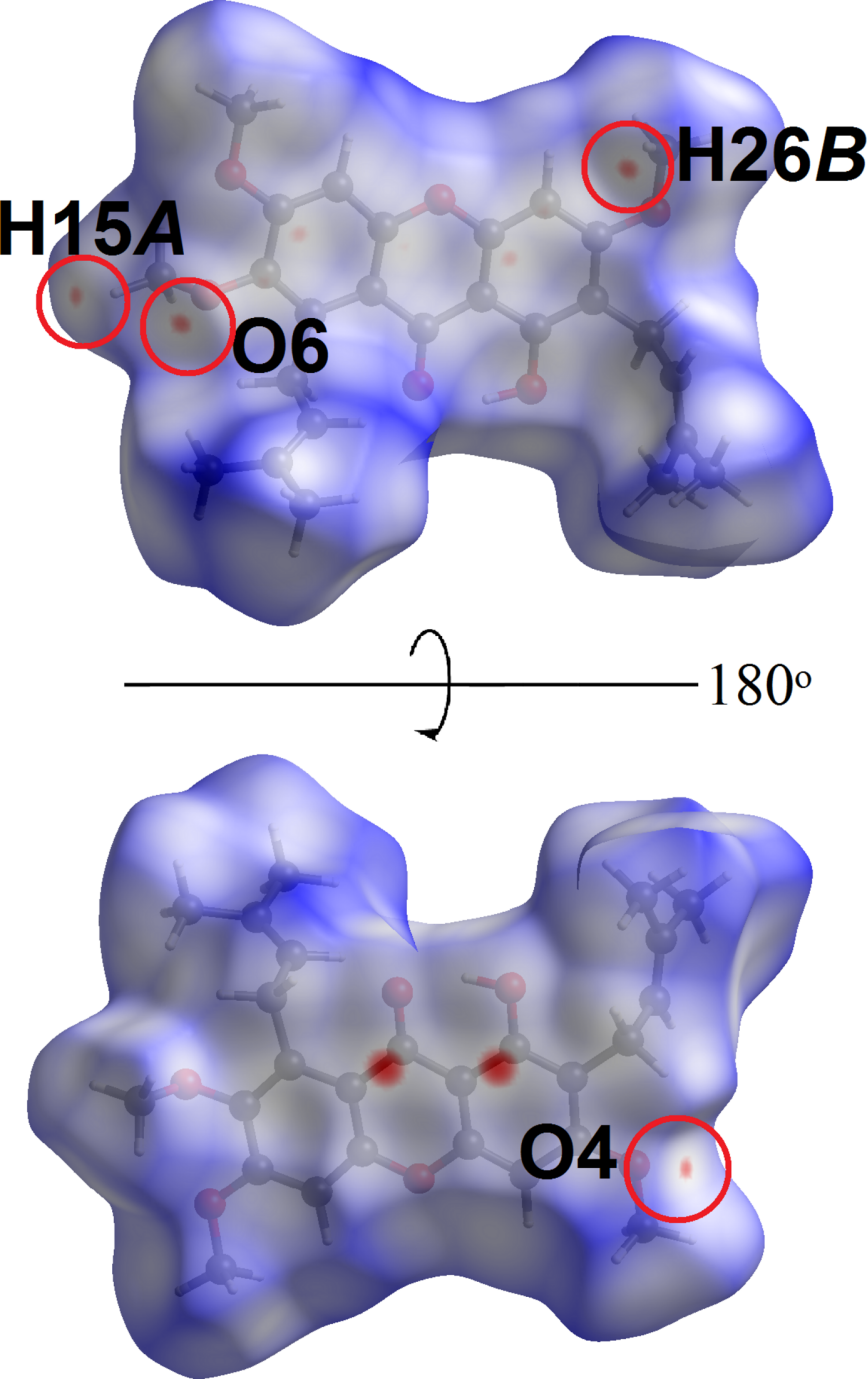
Views of the Hirshfeld surface of (**I**) over *d*_norm_ in the range −0.08 to +1.56 arbitrary units, highlighting C—H⋯O inter­actions.

**Figure 6:**
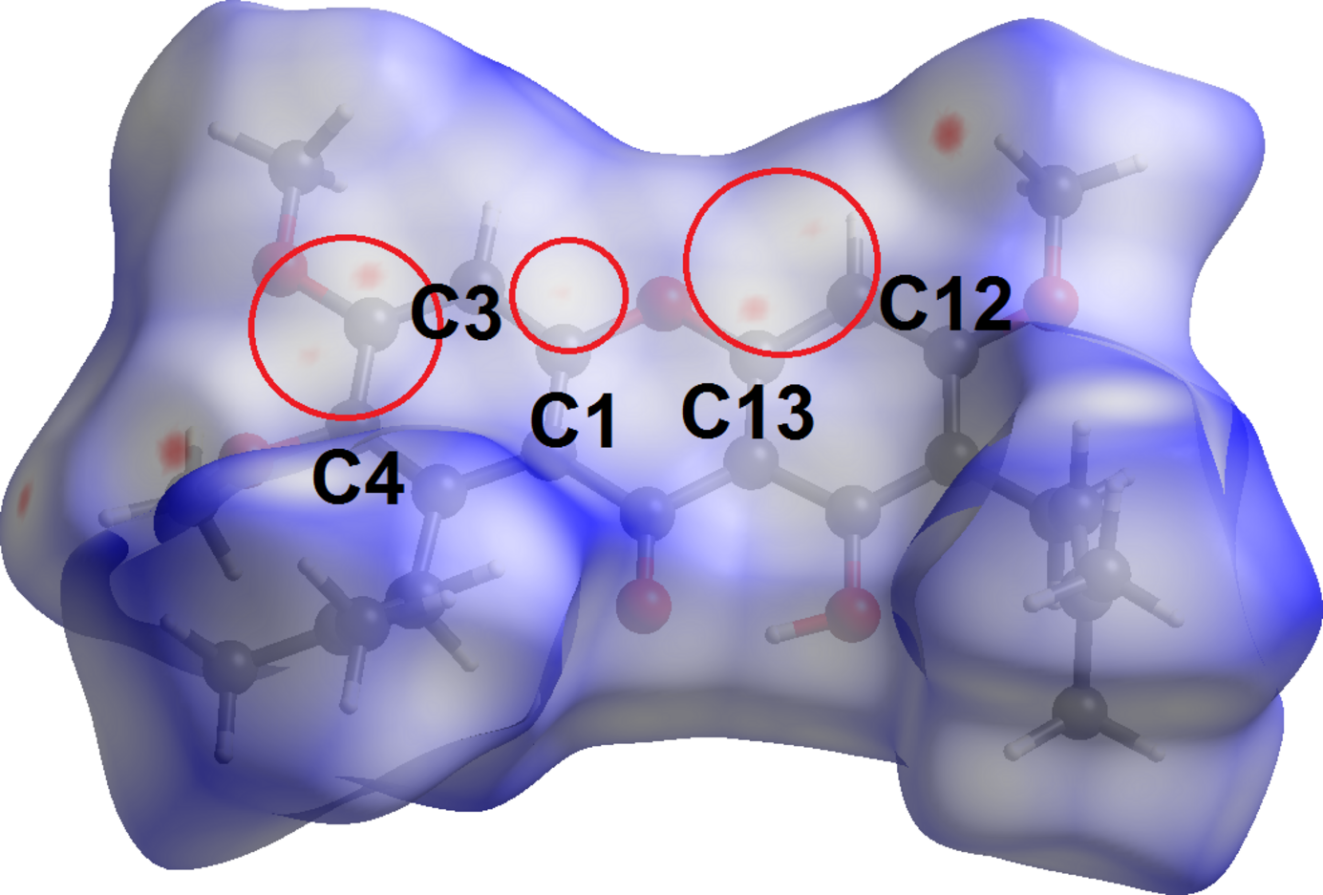
A view of the Hirshfeld surface of (**I**) over *d*_norm_ highlighting C⋯C short contacts in red circles.

In order to acquire further information on the supra­molecular inter­actions between mol­ecules in the crystal of (**I**), the Hirshfeld surface and two-dimensional fingerprint plots were calculated at the HF/STO-3 level of wave function theory by employing the program *Crystal Explorer 17* (Turner *et al.*, 2017 ▸). The bright-red spots on the Hirshfeld surface mapped over *d*_norm_ in Fig. 4 ▸*a*, *i.e.* near the pyrone-C7 and phenol-C9 atoms, correspond to the C7⋯C9 short contacts with separation ∼0.13 Å shorter than the sum of their van der Waals radii, Table 2 ▸. At the same time, the C—H⋯π and ketone⋯π inter­actions are shown as orange ‘potholes’ in the shape index-Hirshfeld surface (Fig. 4 ▸*b*). In Fig. 5 ▸, the faint-red spots appearing near methyl-H15*A*, H26*B* and meth­oxy-O4, O6 atoms correspond to the weak meth­yl⋯meth­oxy inter­action (Table 2 ▸). In addition, the faint-red spots between the overall xanthone ring (Fig. 6 ▸) are correlated to the carbon⋯carbon (C1⋯C1, C3⋯C13 and C4⋯C12) short contacts: these separations are 3.37 Å and 3.39 Å (Table 2 ▸), respectively.

**Figure 7:**
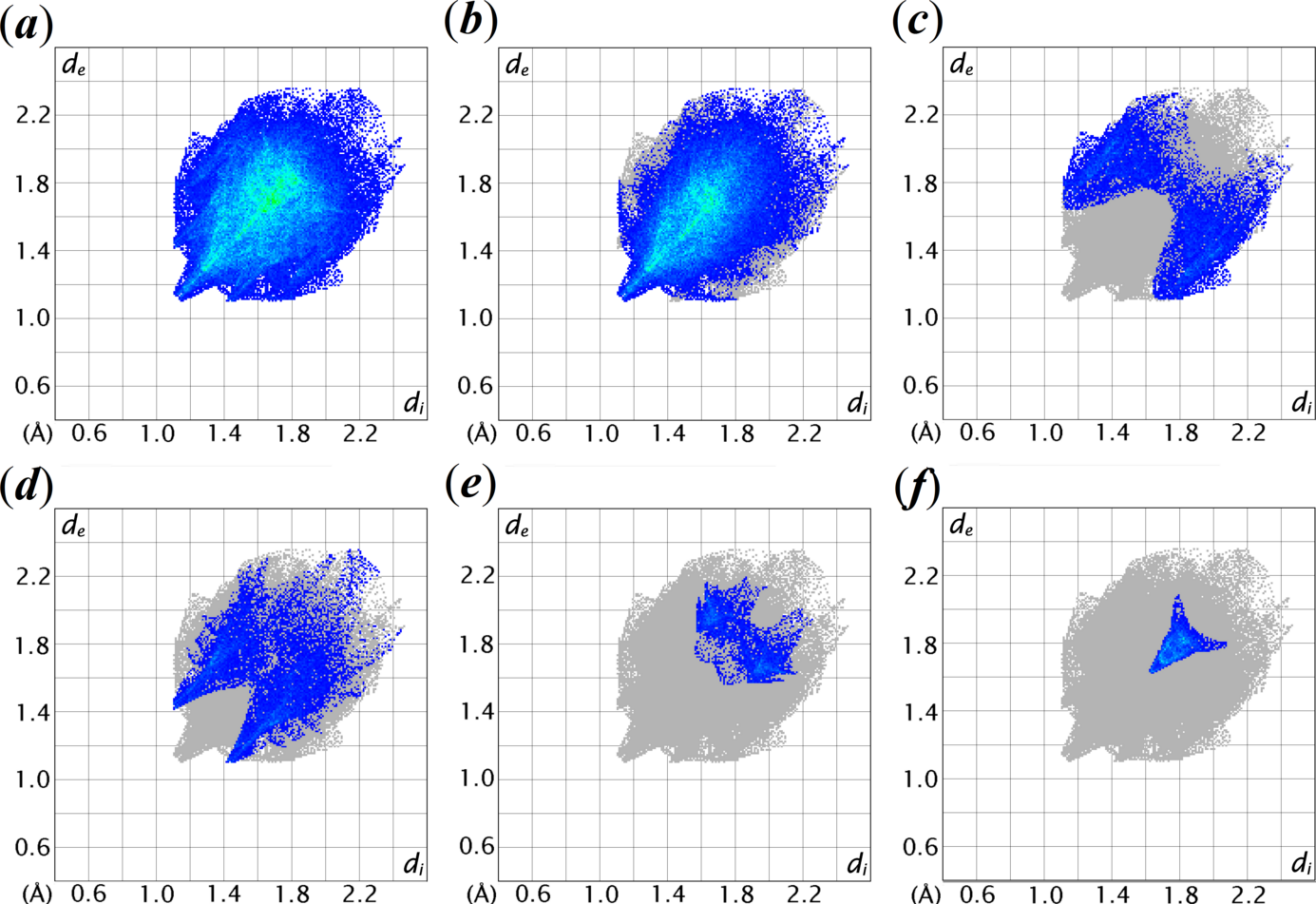
The two-dimensional fingerprint plots of (**I**) for different inter­molecular contacts and their percentage contributions to the Hirshfeld surface.

As illustrated in Fig. 7 ▸, the overall two-dimensional fingerprint plot for the Hirshfeld surface of (**I**) is shown with pseudo-symmetric wings in the upper left and lower right sides of the *d*_e_ and *d*_i_ diagonal axes. The delineated H⋯H, H⋯C/C⋯H, H⋯O/O⋯H, C⋯O/O⋯C and C⋯C contacts are embellished in individual fingerprint plots in Fig. 7 ▸*b*–*f*, respectively. The greatest contribution to the overall Hirshfeld surface is due to H⋯H contacts, which contribute 65.8% and features a beak-shaped peak tipped at *d*_e_ = *d*_i_ ∼2.2 Å. The tip of this H⋯H contact corresponds to a H22*A*⋯H26*A* contact with a distance of 2.25 Å. Consistent with the C—H⋯π and C—H⋯O inter­actions manifested in the mol­ecular packing, H⋯C/C⋯H and H⋯O/O⋯H contacts are the next most prominent contacts, with percentage contributions of 12.9 and 12.3% to the overall Hirshfeld surface. The peak of those contacts tipped at *d*_e_ + *d*_i_ ∼2.8 and 2.5 Å, respectively, as seen in Fig. 7 ▸*c–d*. The C⋯O/O⋯C contacts contribute 5.0% and appears as two blunt-symmetric wings at *d*_e_ + *d*_i_ ∼3.3 Å of the Hirshfeld surface, Fig. 7 ▸*e*. This feature reflects the ketone—O2⋯π inter­action evinced in the mol­ecular packing. The C⋯C contacts contribute 3.8% and features beak-shaped tips at *d*_e_ + *d*_i_ ∼3.2 Å, Fig. 7 ▸*f*; this reflects the C⋯C short contacts between the pyrone and phenol rings. The other inter­atomic contacts have a negligible effect on the mol­ecular packing as it only contributes 0.2% to the overall Hirshfeld surface, Table 3 ▸.

## 5. Energy frameworks

**Figure 8:**
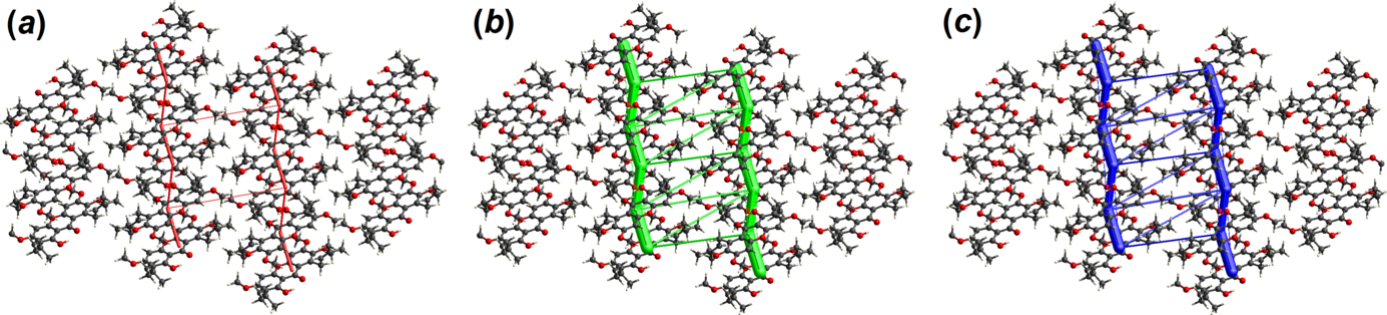
Perspective views of the energy frameworks calculated for (**I**) showing (***a***) electrostatic potential force, (***b***) dispersion force and (***c***) total energy, each plotted down the *b* axis direction. The radii of the cylinders are proportional to the relative magnitudes of the corresponding energies and were adjusted to the same scale factor of 50 with a cut-off value of 5 kJ mol^−1^ within 1 × 1 × 1 unit cells.

The pairwise inter­action energies between the mol­ecules in the crystal of (**I**) were calculated by employing the 6-31G (d,p) basic set with the B3LYP function. The total inter­action energies (*E*_tot_), which comprises the electrostatic (*E*_ele_), polarization (*E*_pol_), dispersion (*E*_dis_) and exchange-repulsion (*E*_rep_) energies were calculated using *Crystal Explorer 17*. The characteristics of the calculated inter­molecular inter­action energies are collated in Table 4 ▸. As anti­cipated, the dispersive component is the major contribution to the inter­action energies owing the absence of conventional hydrogen bonding. The most significant stabilization energies found in the intra-layer region arise from the weak C26—H26*B*⋯O6 inter­action and carbon⋯carbon short contacts (*E*_tot_ = −116.8 kJ mol^−1^). Meanwhile, in the inter-layer region, the most significant stabilization energies arise from the H1*O*1⋯H25*C* and H1*O*3⋯H24*B* contacts (*E*_tot_ = −30.9 kJ mol^−1^). The total *E*_ele_ and *E*_dis_ components of all pairwise inter­action sum to −81.7 and −449.0 kJ mol^−1^, respectively. This observation is also highlighted in the energy framework diagrams, Fig. 8 ▸, as the wider cylinder (greater energy) is shown in the dispersion force.

## 6. Database survey

A search in the Cambridge Structural Database (CSD, version 5.42, last update November 2020; Groom *et al.*, 2016 ▸) using (**I**) as reference structure resulted in three similar structures with different substituents, *i.e*. 1,6-dihy­droxy-3,7-dimeth­oxy-2,8-bis­(3-methyl­but-2-en-1-yl)-9H-xanthen-9-one (CSD refcode QAYQAJ; Chantrapromma *et al.*, 2006 ▸), 7-meth­oxy-2,8-bis­(3-methyl­but-2-en-1-yl)-9-oxo-9*H*-xanthene-1,3,6-triyl tri­acetate (VUYLUW; Ravikumar *et al.*, 1988 ▸) and 1,3,6-trihy­droxy-7-meth­oxy-2,8-bis­(3-methyl­but-2-en-1-yl)-9*H*-xanthen-9-one (WAFVAC; Ee *et al.*, 2010 ▸). Details of the selected dihedral and torsion angles for the bis­(methyl­buten­yl) xanthenone moiety in these structures are listed in Table 5 ▸. By analogy with (**I**), the central pyrone ring systems are almost planar with the dihedral angles between the phenyl rings in the range of 0.33–5.46°. Both phenyl rings are less coplanar with each other as compared to dihedral angles 1 and 2, especially in VUYLUM where its dihedral angle 3 is 8.90°. The torsion angle between the phenyl ring and the ethyl moiety (C4—C5—C16—C17, τ1 and C9—C10—C21—C22, τ5; our atom-numbering scheme) are either in *syn*-clinal (67.6–87.5°) or *anti*-clinal (98.57–103.18°) conformations. The torsion angles between the ethyl and ethene moiety (C5—C16—C17—C18, τ2 and C10—C21—C22—C23, τ6) are all in anti-clinal conformations with a range of 97.8–142.7°. As expected for the *sp*^2^ hybridized atoms of the butenyl moiety, the attached methyl moieties are in-plane with the ethene moiety. This is indicated by the torsion angles τ3 (C16—C17—C18—C19) and τ7 (C21—C22—C23—C24) = 0.3–3.8° and τ4 (C16—C17—C18—C20) and τ8 (C21—C22—C23—C25) = 176.0–179.5°. Besides the 2,8-bis­(methyl­buten­yl) xanthenone moiety, there are two structures (ILUCOH; Pettit *et al.*, 2003 ▸) and (ILUCOH01; Buitrago Díaz *et al.*, 2010 ▸) containing an 8-methyl xanthenone moiety.

## 7. Isolation and crystallization

The stem bark of *Cratoxylum glaucum* (3.75 kg) was air dried and ground into powder. It was then subjected to solvent extraction using distilled *n*-hexane for 72 h. The resulting *n*-hexane solution was then filtered and the bark sample was then re-extracted twice with fresh portions of *n*-hexane. The three *n*-hexane solutions were combined, concentrated with a rotary evaporator under reduced pressure to result in 25.6 g of dry crude extract. The *n*-hexane crude extract (18.0 g) was chromatographed using a vacuum column packed with silica gel (Merck 7731) and eluted with *n*-hexane, hexa­ne/chloro­form, chloro­form/ethyl acetate, ethyl acetate/methanol and methanol/acetone stepwise with increasing polarity to yield 25 fractions. These fractions were monitored with TLC plates. Fractions with similar compositions were combined. Fractions 8, 9 and 10 were further chromatographed in a smaller gravity silica gel column and eluted with *n*-hexane, hexa­ne/chloro­form, chloro­form/ethyl acetate and ethyl acetate/methanol stepwise with increasing polarity. The sub-fractions were checked using TLC plates. The chloro­form subfractions were combined, dried and washed with methanol. The clean fraction was then dissolved in chloro­form and yellow crystals of (**I**) were successfully recrystallized.

These yellow crystals have a melting point of 387–388 K (Lit. 383–389 K; Yates & Stout, 1958 ▸) with an *R*_f_ value of 0.62 on an analytical TLC plate with 100% chloro­form as the mobile phase. The UV maximum absorption exhibited the characteristic absorption bands of a xanthone ring system at 247, 264, 313 and 352 nm. The IR spectrum of the crystals showed evidence of the existence of a hydroxyl group, CH_3_ and CH_2_ groups, a conjugated C=O, C=C (aromatic), C=C (alkene) group as well as a C—O (ether) groups. These data together with the detailed structural elucidation using two-dimensional NMR techniques have led to the mol­ecular structure of fuscaxantone C, which is also in agreement with the mol­ecular ion peak at *m*/*z* 438 in the mass spectrum.

UV(EtOH) λ_max_ nm (log ɛ): 247 (4.40), 264 (4.47), 313 (4.30), 352 (3.75). IR ν_max_ cm^−1^ (UATR): 3436 (OH stretch), 2964 (CH_3_ stretch), 2922 (CH_2_ stretch), 1646 (C=O stretch), 1605 (C=C stretch), 1462 (C=C aromatic), 1216 (C—O), 896–702 (oop bending). EI-MS *m*/*z* (ret. int.): 438 *M*^+^ (71), 423 (8), 395 (75), 372 (65), 367 (100), 351 (43), 339 (57), 313 (18). ^1^H NMR (400 MHz, CDCl_3_): *δ* 13.46 (*s*, 1H, 9-OH), 6.67 (*s*, 1H, H-2), 6.25 (*s*, 1H, H-12), 5.24 (*m*, H-17), 5.23 (*m*, H-22), 4.10 (*d*, 2H, *J* = 6.9 Hz, H-16), 3.93 (*s*, 3H, H-14), 3.87 (*s*, 3H, H-26), 3.78 (*s*, 3H, H-15), 3.31 (*d*, 2H, *J* = 7.4 Hz, H-21), 1.85 (*s*, 3H, H-20), 1.80 (*s*, 3H, H-25), 1.67 (*s*, 6H, H-19 & H-24). ^13^C NMR (100 MHz, CDCl_3_): *δ* 181.9 (C-7), 163.3 (C-11), 159.6 (C-9), 157.9 (C-3), 155.2 (C-1), 155.0 (C-13), 143.9 (C-4), 137.0 (C-5), 131.6 (C-23), 131.6 (C-18), 123.3 (C-17), 122.3 (C-22), 111.9 (C-6), 111.3 (C-10), 103.8 (C-8), 98.1 (C-2), 88.5 (C-12), 60.8 (C-15), 55.9 (C-14), 55.7 (C-26), 26.1 (C-16), 25.9 (C-19), 25.9 (C-24), 25.8 (C-19) 21.3 (C-21), 18.1 (C-20), 17.7 (C-25)

**Structure elucidation description**

The ^1^H NMR spectrum of (**I**) clearly indicated the presence of three methoxyl groups which appeared as a three-hydrogen singlet each at *δ* 3.87, 3.93 and 3.78. A low field hydroxyl group was observed at *δ* 13.46. Two aromatic proton singlets were observed at *δ* 6.25 and 6.67 and were assigned to H-4 and H-5, respectively. Meanwhile, the coupling between a multiplet at *δ* 5.24 and H-21 (*δ* 3.31) and H-16 (*δ* 4.10) at their respective prenyl moiety was observed in the COSY spectrum. Thus, this multiplet which consists of two protons was assigned to be H-22 and H-17.

The ^13^C NMR spectrum revealed the existence of 26 carbon atoms. Signals for conjugated carbonyl and three methoxyl groups were observed at *δ* 181.9 (C-7), 55.7 (C-26), 55.9 (C-14) and 60.8 (C-15), respectively. The ^13^C NMR signal at *δ* 159.6 (C-9), 163.3 (C-11), 157.9 (C-3) and 143.9 (C-4) showed the presence of four oxygenated carbon atoms in the xanthone skeleton. The DEPT spectrum indicated that four CH groups, two CH_2_ groups, three methoxyl group and four CH_3_ were present in the mol­ecular structure.

From the HMQC experiment, the linkage of the two protons, H-12 (*δ* 6.25) and H-2 (*δ* 6.67), to their respective unsubstituted aromatic carbon atoms, C-12 (*δ* 88.5) and C-2 (*δ* 98.1), were observed. Meanwhile, the three methoxyl groups were linked to the assigned carbon atoms at *δ* 55.7 (C-11), 55.9 (C-3) and 60.8 (C-4). The HMBC spectrum indicated the linkage of the chelated hydroxyl group to carbon atoms at *δ* 103.8 (C-8), 111.3 (C-10) and 159.6 (C-9). This confirmed the position of the hydroxyl group at C-9 (*δ* 159.6). The positions of the three methoxyl groups were confirmed through the evidence of HMBC linkage of proton signals at *δ* 3.87 (C-26), 3.93 (C-14) and 3.78 (C-15) to their respective aromatic carbon atoms in the xanthone skeleton at C-11 (*δ* 163.3), C-3 (*δ* 157.9) and C-4 (*δ* 143.9).

## 8. Refinement

Crystal data, data collection and structure refinement details are summarized in Table 6 ▸. C-bound H atoms were positioned geometrically [C—H = 0.93–0.97 Å] and refined using a riding model with *U*_iso_(H) = 1.2*U*_eq_(C) or 1.5*U*_eq_(C–meth­yl). The O-bound hydrogen atom was located from difference-Fourier maps and refined freely.

## Supplementary Material

Crystal structure: contains datablock(s) I. DOI: 10.1107/S2056989026004792/hb8201sup1.cif

Structure factors: contains datablock(s) I. DOI: 10.1107/S2056989026004792/hb8201Isup3.hkl

Supporting information file. DOI: 10.1107/S2056989026004792/hb8201Isup3.cml

CCDC reference: 2553254

Additional supporting information:  crystallographic information; 3D view; checkCIF report

## Figures and Tables

**Table 1:** Hydrogen-bond geometry (Å, °) *Cg*1 is the centroid of the O1/C1/C6–C8/C13 ring.

*D*—H⋯*A*	*D*—H	H⋯*A*	*D*⋯*A*	*D*—H⋯*A*
O3—H1*O*3⋯O2	0.86 (2)	1.75 (2)	2.5518 (14)	155 (2)
C26—H26*B*⋯O6^i^	0.96	2.64	3.509 (2)	151
C15—H15*A*⋯O4^ii^	0.96	2.66	3.554 (2)	156
C21—H21*A*⋯*Cg*1^iii^	0.97	2.84	3.6949 (15)	148

Symmetry codes: (i) 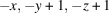; (ii) 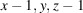; (iii) 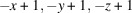.

**Table 2:** Summary of short inter­atomic contacts (Å) in (**I**)^*a*^

Contact	Distance	Symmetry operation
C26—H26*B*⋯O6^*b*^	2.53	−*x*, −*y* + 1, −*z* + 1
C15—H15*A*⋯O4^*b*^	2.55	*x* − 1, *y*, *z* − 1
C7⋯C9	3.27	−*x* + 1, −*y* + 1, −*z* + 1
C1⋯C1	3.39	−*x*, −*y* + 1, −*z* + 1
C3⋯C13	3.37	−*x*, −*y* + 1, −*z* + 1
C4⋯C12	3.39	−*x*, −*y* + 1, −*z* + 1

Notes: (*a*) The inter­atomic distances are measured in *Crystal Explorer 17* whereby the *X*—H bond lengths are adjusted to their neutron values; (*b*) these inter­actions correspond to the inter­action listed in Table 1 ▸.

**Table 3:** Percentage contributions of inter­atomic contacts to the Hirshfeld surface of (**I**)

Contact	Percentage contribution
H⋯H	65.8
H⋯C/C⋯H	12.9
H⋯O/O⋯H	12.3
O⋯C/C⋯O	5.0
C⋯C	3.8
O⋯O	0.2

**Table 4:** A summary of inter­action energies (kJ mol^−1^) calculated for (**I**)

Contact	*R* (Å)	*E*_ele_	*E*_pol_	*E*_dis_	*E*_rep_	*E*_tot_
H19*C*⋯H20*B*	12.56	–2.2	–0.7	–17.0	6.5	–13.6
C15—H15*A*⋯O4	14.01	–3.5	–1.3	–22.8	13.4	–16.2
H20*C*⋯H26*C*	14.71	–0.7	–0.2	–5.0	1.8	–4.1
H15*C*⋯H20*B*	10.23	–2.9	–0.5	–19.5	7.6	–15.8
H19*B*⋯H20*C*	15.73	0.0	0.0	–1.8	0.0	–1.5
H20*C*⋯H25*A*	9.53	–0.9	–0.3	–13.0	7.8	–7.6
H15*B*⋯H19*A*	12.63	–4.0	–0.7	–22.2	12.4	–16.4
C26—H26*B*⋯O6+C1⋯C1+C3⋯C13+C4⋯C12	4.70	–34.1	–10.4	–146.7	88.6	–116.8
H14*C*⋯H24*B*	14.22	–3.0	–0.5	–5.9	2.6	–7.0
H14*B*⋯H14*B*	12.75	2.6	–1.2	–8.7	4.0	–3.2
H1*O*1⋯C25*C*+H1*O*3⋯H24*B*	10.37	–6.7	–2.6	–44.2	26.9	–30.9
C7⋯C9	4.98	–18.1	–3.4	–120.6	69.8	–83.5
H22*A*⋯H26*A*	13.18	–8.2	–1.6	–21.6	13.2	–20.6

**Table 5:** Selected dihedral and torsion angles (°) Dihedral angles 1 and 2 are the angles between the mean planes of the O1/C1/C6–C8/C13 pyrone ring and the C1–C6 and C8–C13 phenyl rings, respectively. Dihedral angle 3 is the angle between the mean planes of C1–C6 and C8–C13 phenyl rings (our atom-numbering scheme).

	(**I**)	QAYQAJ	VUYLUM	WAFVAC
Dihedral angle 1	1.32 (6)	3.65	5.46	1.62
Dihedral angle 2	3.56 (6)	0.33	3.55	1.69
Dihedral angle 3	4.63 (6)	3.97	8.90	1.72
τ1 (C4—C5—C16—C17)	–101.46 (14)	98.6	–103.2	80.3
τ2 (C5—C16—C17—C18)	125.59 (16)	−103.7	142.7	–122.7
τ3 (C16—C17—C18—C19)	–1.4 (3)	–3.8	–2.0	–2.3
τ4 (C16—C17—C18—C20)	177.62 (15)	176.5	176.0	176.6
τ5 (C9—C10—C21—C22)	–78.58 (16)	100.4	–67.6	–87.4
τ6 (C10—C21—C22—C23)	120.37 (15)	–105.0	–97.8	–123.1
τ7 (C21—C22—C23—C24)	–0.3 (2)	2.3	–1.0	1.0
τ8 (C21—C22—C23—C25)	–179.48 (13)	–178.5	179.16	–179.5

**Table 6:** Experimental details

Crystal data	
Chemical formula	C_26_H_30_O_6_
*M*_r_	438.50
Crystal system, space group	Triclinic, *P* 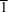
Temperature (K)	150
*a*, *b*, *c* (Å)	9.1407 (7), 10.2279 (10), 13.1741 (12)
α, β, γ (°)	105.157 (8), 104.604 (7), 94.347 (7)
*V* (Å^3^)	1137.07 (18)
*Z*	2
Radiation type	Cu *K*α
μ (mm^−1^)	0.74
Crystal size (mm)	0.25 × 0.17 × 0.07

Data collection	
Diffractometer	Xcalibur, Eos, Gemini
Absorption correction	Multi-scan (*CrysAlis PRO*; Agilent, 2012 ▸)
*T*_min_, *T*_max_	0.920, 1.000
No. of measured, independent and observed [*I* > 2σ(*I*)] reflections	7762, 4127, 3295
*R*_int_	0.017
(sin θ/λ)_max_ (Å^−1^)	0.606

Refinement	
*R*[*F*^2^ > 2σ(*F*^2^)], *wR*(*F*^2^), *S*	0.040, 0.115, 1.06
No. of reflections	4127
No. of parameters	300
H-atom treatment	H atoms treated by a mixture of independent and constrained refinement
Δρ_max_, Δρ_min_ (e Å^−3^)	0.28, −0.18

Computer programs: *CrysAlis PRO* (Agilent, 2012 ▸), *SHELXT* (Sheldrick, 2015*a* ▸), *SHELXL2018/3* (Sheldrick, 2015*b* ▸), *Mercury* (Macrae *et al.*, 2020 ▸) and *publCIF* (Westrip, 2010 ▸).

## supplementary crystallographic information

### 1-Hydroxy-3,6,7-trimethoxy-2,8-bis(3-methylbut-2-en-1-yl)-9H-xanthen-9-one Crystal data

**Table:**

C_26_H_30_O_6_	*Z* = 2
*M**_r_* = 438.50	*F*(000) = 468
Triclinic, *P*1	*D*_x_ = 1.281 Mg m^−^^3^
*a* = 9.1407 (7) Å	Cu *K*α radiation, λ = 1.54178 Å
*b* = 10.2279 (10) Å	Cell parameters from 5280 reflections
*c* = 13.1741 (12) Å	θ = 3.6–71.5°
α = 105.157 (8)°	µ = 0.74 mm^−^^1^
β = 104.604 (7)°	*T* = 150 K
γ = 94.347 (7)°	Prismatic, yellow
*V* = 1137.07 (18) Å^3^	0.25 × 0.17 × 0.07 mm

### 1-Hydroxy-3,6,7-trimethoxy-2,8-bis(3-methylbut-2-en-1-yl)-9H-xanthen-9-one Data collection

**Table:**

Xcalibur, Eos, Gemini diffractometer	4127 independent reflections
Radiation source: fine-focus sealed X-ray tube, Enhance (Cu) X-ray Source	3295 reflections with *I* > 2σ(*I*)
Graphite monochromator	*R*_int_ = 0.017
Detector resolution: 16.1952 pixels mm^-1^	θ_max_ = 69.0°, θ_min_ = 3.6°
ω scans	*h* = −10→11
Absorption correction: multi-scan (CrysAlisPro; Agilent, 2012)	*k* = −11→12
*T*_min_ = 0.920, *T*_max_ = 1.000	*l* = −14→15
7762 measured reflections	

### 1-Hydroxy-3,6,7-trimethoxy-2,8-bis(3-methylbut-2-en-1-yl)-9H-xanthen-9-one Refinement

**Table:**

Refinement on *F*^2^	0 restraints
Least-squares matrix: full	Hydrogen site location: mixed
*R*[*F*^2^ > 2σ(*F*^2^)] = 0.040	H atoms treated by a mixture of independent and constrained refinement
*wR*(*F*^2^) = 0.115	*w* = 1/[σ^2^(*F*_o_^2^) + (0.0772*P*)^2^ + 0.0205*P*] where *P* = (*F*_o_^2^ + 2*F*_c_^2^)/3
*S* = 1.06	(Δ/σ)_max_ < 0.001
4127 reflections	Δρ_max_ = 0.28 e Å^−^^3^
300 parameters	Δρ_min_ = −0.17 e Å^−^^3^

### 1-Hydroxy-3,6,7-trimethoxy-2,8-bis(3-methylbut-2-en-1-yl)-9H-xanthen-9-one Special details

**Table:**

Geometry. All esds (except the esd in the dihedral angle between two l.s. planes) are estimated using the full covariance matrix. The cell esds are taken into account individually in the estimation of esds in distances, angles and torsion angles; correlations between esds in cell parameters are only used when they are defined by crystal symmetry. An approximate (isotropic) treatment of cell esds is used for estimating esds involving l.s. planes.

### 1-Hydroxy-3,6,7-trimethoxy-2,8-bis(3-methylbut-2-en-1-yl)-9H-xanthen-9-one Fractional atomic coordinates and isotropic or equivalent isotropic displacement parameters (Å^2^)

**Table:**

	*x*	*y*	*z*	*U*_iso_*/*U*_eq_	
O1	0.20864 (10)	0.64184 (9)	0.58521 (7)	0.0281 (2)	
O2	0.29926 (11)	0.30741 (10)	0.37232 (8)	0.0343 (2)	
O3	0.50729 (11)	0.27730 (9)	0.53122 (8)	0.0317 (2)	
H1O3	0.451 (2)	0.271 (2)	0.4667 (17)	0.053 (5)*	
O4	0.55801 (11)	0.55703 (10)	0.88630 (8)	0.0336 (2)	
O5	−0.16757 (11)	0.73855 (10)	0.30739 (8)	0.0342 (2)	
O6	−0.13561 (11)	0.52091 (10)	0.15539 (8)	0.0329 (2)	
C1	0.13141 (14)	0.60507 (13)	0.47582 (10)	0.0263 (3)	
C2	0.02684 (14)	0.69132 (13)	0.44998 (11)	0.0277 (3)	
H2A	0.015963	0.766348	0.503857	0.033*	
C3	−0.06070 (14)	0.66322 (13)	0.34249 (11)	0.0281 (3)	
C4	−0.04234 (14)	0.54908 (14)	0.26131 (11)	0.0276 (3)	
C5	0.06237 (14)	0.46339 (13)	0.28679 (11)	0.0269 (3)	
C6	0.15297 (14)	0.49124 (13)	0.39779 (10)	0.0261 (3)	
C7	0.27032 (15)	0.41048 (13)	0.43516 (11)	0.0270 (3)	
C8	0.35072 (14)	0.45502 (13)	0.55089 (10)	0.0262 (3)	
C9	0.46553 (15)	0.38372 (13)	0.59729 (11)	0.0268 (3)	
C10	0.53498 (14)	0.41965 (13)	0.70923 (11)	0.0273 (3)	
C11	0.48919 (14)	0.52991 (13)	0.77695 (10)	0.0273 (3)	
C12	0.38124 (15)	0.60555 (13)	0.73502 (10)	0.0270 (3)	
H12A	0.354567	0.679949	0.780831	0.032*	
C13	0.31536 (14)	0.56612 (13)	0.62314 (11)	0.0258 (3)	
C14	−0.18395 (17)	0.85795 (15)	0.38651 (13)	0.0397 (3)	
H14A	−0.265105	0.900649	0.352897	0.060*	
H14B	−0.090107	0.921109	0.413474	0.060*	
H14C	−0.207647	0.832339	0.446306	0.060*	
C15	−0.09384 (18)	0.61182 (16)	0.09751 (12)	0.0386 (3)	
H15A	−0.166548	0.590806	0.026559	0.058*	
H15B	0.006192	0.600776	0.089063	0.058*	
H15C	−0.093197	0.704751	0.138153	0.058*	
C16	0.07759 (16)	0.34501 (13)	0.19452 (10)	0.0293 (3)	
H16A	0.036418	0.363546	0.125148	0.035*	
H16B	0.185152	0.338583	0.203267	0.035*	
C17	−0.00433 (17)	0.20957 (14)	0.19140 (11)	0.0341 (3)	
H17A	0.021282	0.184542	0.255902	0.041*	
C18	−0.10879 (17)	0.12212 (14)	0.10703 (12)	0.0349 (3)	
C19	−0.1690 (2)	0.14327 (18)	−0.00325 (13)	0.0501 (4)	
H19A	−0.135150	0.235952	0.000286	0.075*	
H19B	−0.278752	0.126109	−0.024836	0.075*	
H19C	−0.131690	0.081325	−0.055935	0.075*	
C20	−0.1764 (2)	−0.01267 (17)	0.11535 (16)	0.0564 (5)	
H20A	−0.128684	−0.022371	0.186220	0.085*	
H20B	−0.159271	−0.086563	0.059560	0.085*	
H20C	−0.284373	−0.014804	0.105557	0.085*	
C21	0.65314 (15)	0.33840 (13)	0.75469 (11)	0.0305 (3)	
H21A	0.720322	0.319562	0.707908	0.037*	
H21B	0.714818	0.393030	0.827021	0.037*	
C22	0.58187 (15)	0.20484 (14)	0.76256 (10)	0.0289 (3)	
H22A	0.515991	0.211114	0.806548	0.035*	
C23	0.60252 (16)	0.07913 (14)	0.71376 (11)	0.0318 (3)	
C24	0.7034 (2)	0.04698 (16)	0.63916 (15)	0.0472 (4)	
H24A	0.749913	0.130726	0.632793	0.071*	
H24B	0.643148	−0.009169	0.568041	0.071*	
H24C	0.781706	−0.001164	0.668998	0.071*	
C25	0.52150 (19)	−0.04507 (15)	0.72913 (14)	0.0430 (4)	
H25A	0.467327	−0.016348	0.782814	0.064*	
H25B	0.595142	−0.100412	0.753662	0.064*	
H25C	0.450412	−0.097586	0.660723	0.064*	
C26	0.50825 (17)	0.66040 (16)	0.96059 (11)	0.0376 (3)	
H26A	0.559934	0.663895	1.034583	0.056*	
H26B	0.399876	0.639155	0.947968	0.056*	
H26C	0.531490	0.747647	0.949176	0.056*	

### 1-Hydroxy-3,6,7-trimethoxy-2,8-bis(3-methylbut-2-en-1-yl)-9H-xanthen-9-one Atomic displacement parameters (Å^2^)

**Table:**

	*U*^11^	*U*^22^	*U*^33^	*U*^12^	*U*^13^	*U*^23^
O1	0.0287 (5)	0.0278 (5)	0.0259 (5)	0.0075 (4)	0.0043 (4)	0.0071 (4)
O2	0.0370 (5)	0.0314 (5)	0.0291 (5)	0.0110 (4)	0.0038 (4)	0.0034 (4)
O3	0.0323 (5)	0.0293 (5)	0.0299 (5)	0.0100 (4)	0.0039 (4)	0.0058 (4)
O4	0.0359 (5)	0.0368 (5)	0.0249 (5)	0.0091 (4)	0.0033 (4)	0.0078 (4)
O5	0.0284 (5)	0.0368 (5)	0.0341 (5)	0.0106 (4)	0.0015 (4)	0.0102 (4)
O6	0.0288 (5)	0.0377 (5)	0.0288 (5)	0.0019 (4)	0.0011 (4)	0.0118 (4)
C1	0.0243 (6)	0.0274 (6)	0.0261 (6)	0.0008 (5)	0.0047 (5)	0.0094 (5)
C2	0.0264 (6)	0.0259 (6)	0.0299 (7)	0.0040 (5)	0.0074 (5)	0.0070 (5)
C3	0.0219 (6)	0.0294 (7)	0.0336 (7)	0.0014 (5)	0.0054 (5)	0.0135 (5)
C4	0.0223 (6)	0.0313 (7)	0.0271 (6)	−0.0016 (5)	0.0030 (5)	0.0106 (5)
C5	0.0260 (6)	0.0259 (6)	0.0287 (7)	−0.0010 (5)	0.0069 (5)	0.0102 (5)
C6	0.0248 (6)	0.0255 (6)	0.0282 (7)	0.0011 (5)	0.0067 (5)	0.0098 (5)
C7	0.0273 (6)	0.0245 (6)	0.0291 (7)	0.0019 (5)	0.0081 (5)	0.0078 (5)
C8	0.0249 (6)	0.0249 (6)	0.0283 (7)	0.0012 (5)	0.0065 (5)	0.0087 (5)
C9	0.0256 (6)	0.0231 (6)	0.0309 (7)	0.0013 (5)	0.0074 (5)	0.0077 (5)
C10	0.0241 (6)	0.0256 (6)	0.0312 (7)	0.0016 (5)	0.0049 (5)	0.0099 (5)
C11	0.0257 (6)	0.0280 (6)	0.0256 (6)	−0.0010 (5)	0.0031 (5)	0.0088 (5)
C12	0.0287 (6)	0.0240 (6)	0.0271 (7)	0.0030 (5)	0.0077 (5)	0.0056 (5)
C13	0.0238 (6)	0.0243 (6)	0.0301 (7)	0.0028 (5)	0.0057 (5)	0.0114 (5)
C14	0.0346 (7)	0.0367 (8)	0.0412 (8)	0.0130 (6)	0.0007 (6)	0.0075 (6)
C15	0.0409 (8)	0.0455 (8)	0.0317 (7)	0.0105 (6)	0.0056 (6)	0.0184 (6)
C16	0.0317 (7)	0.0294 (7)	0.0244 (6)	0.0022 (5)	0.0043 (5)	0.0079 (5)
C17	0.0433 (8)	0.0291 (7)	0.0299 (7)	0.0057 (6)	0.0073 (6)	0.0114 (5)
C18	0.0349 (7)	0.0296 (7)	0.0371 (8)	0.0051 (5)	0.0087 (6)	0.0057 (6)
C19	0.0496 (10)	0.0487 (9)	0.0380 (9)	−0.0004 (7)	−0.0033 (7)	0.0061 (7)
C20	0.0648 (12)	0.0370 (9)	0.0598 (11)	−0.0076 (8)	0.0160 (9)	0.0070 (8)
C21	0.0274 (6)	0.0287 (7)	0.0311 (7)	0.0040 (5)	0.0015 (5)	0.0080 (5)
C22	0.0287 (6)	0.0324 (7)	0.0257 (6)	0.0073 (5)	0.0048 (5)	0.0107 (5)
C23	0.0316 (7)	0.0313 (7)	0.0334 (7)	0.0073 (5)	0.0078 (5)	0.0116 (5)
C24	0.0519 (9)	0.0353 (8)	0.0625 (11)	0.0119 (7)	0.0308 (8)	0.0125 (7)
C25	0.0498 (9)	0.0315 (8)	0.0532 (9)	0.0087 (6)	0.0219 (7)	0.0139 (7)
C26	0.0394 (8)	0.0424 (8)	0.0256 (7)	0.0086 (6)	0.0041 (6)	0.0046 (6)

### 1-Hydroxy-3,6,7-trimethoxy-2,8-bis(3-methylbut-2-en-1-yl)-9H-xanthen-9-one Geometric parameters (Å, º)

**Table:**

O1—C13	1.3682 (15)	C15—H15A	0.9600
O1—C1	1.3712 (15)	C15—H15B	0.9600
O2—C7	1.2501 (16)	C15—H15C	0.9600
O3—C9	1.3502 (16)	C16—C17	1.5095 (18)
O3—H1O3	0.86 (2)	C16—H16A	0.9700
O4—C11	1.3622 (15)	C16—H16B	0.9700
O4—C26	1.4279 (17)	C17—C18	1.328 (2)
O5—C3	1.3544 (16)	C17—H17A	0.9300
O5—C14	1.4286 (17)	C18—C19	1.496 (2)
O6—C4	1.3819 (15)	C18—C20	1.510 (2)
O6—C15	1.4337 (17)	C19—H19A	0.9600
C1—C2	1.3886 (18)	C19—H19B	0.9600
C1—C6	1.4008 (19)	C19—H19C	0.9600
C2—C3	1.3817 (19)	C20—H20A	0.9600
C2—H2A	0.9300	C20—H20B	0.9600
C3—C4	1.4149 (19)	C20—H20C	0.9600
C4—C5	1.3842 (19)	C21—C22	1.5082 (18)
C5—C6	1.4287 (18)	C21—H21A	0.9700
C5—C16	1.5179 (18)	C21—H21B	0.9700
C6—C7	1.4684 (18)	C22—C23	1.3296 (19)
C7—C8	1.4467 (18)	C22—H22A	0.9300
C8—C13	1.3953 (19)	C23—C24	1.503 (2)
C8—C9	1.4256 (18)	C23—C25	1.5070 (19)
C9—C10	1.3864 (19)	C24—H24A	0.9600
C10—C11	1.4059 (19)	C24—H24B	0.9600
C10—C21	1.5119 (17)	C24—H24C	0.9600
C11—C12	1.3934 (18)	C25—H25A	0.9600
C12—C13	1.3794 (18)	C25—H25B	0.9600
C12—H12A	0.9300	C25—H25C	0.9600
C14—H14A	0.9600	C26—H26A	0.9600
C14—H14B	0.9600	C26—H26B	0.9600
C14—H14C	0.9600	C26—H26C	0.9600
			
C13—O1—C1	119.73 (10)	H15B—C15—H15C	109.5
C9—O3—H1O3	104.2 (14)	C17—C16—C5	112.79 (11)
C11—O4—C26	118.13 (10)	C17—C16—H16A	109.0
C3—O5—C14	117.10 (10)	C5—C16—H16A	109.0
C4—O6—C15	113.53 (10)	C17—C16—H16B	109.0
O1—C1—C2	113.02 (11)	C5—C16—H16B	109.0
O1—C1—C6	123.94 (11)	H16A—C16—H16B	107.8
C2—C1—C6	123.03 (12)	C18—C17—C16	127.38 (12)
C3—C2—C1	118.55 (12)	C18—C17—H17A	116.3
C3—C2—H2A	120.7	C16—C17—H17A	116.3
C1—C2—H2A	120.7	C17—C18—C19	125.17 (14)
O5—C3—C2	124.09 (13)	C17—C18—C20	121.19 (14)
O5—C3—C4	115.82 (11)	C19—C18—C20	113.64 (14)
C2—C3—C4	120.09 (12)	C18—C19—H19A	109.5
O6—C4—C5	119.89 (12)	C18—C19—H19B	109.5
O6—C4—C3	118.62 (12)	H19A—C19—H19B	109.5
C5—C4—C3	121.45 (12)	C18—C19—H19C	109.5
C4—C5—C6	118.88 (12)	H19A—C19—H19C	109.5
C4—C5—C16	118.20 (11)	H19B—C19—H19C	109.5
C6—C5—C16	122.91 (12)	C18—C20—H20A	109.5
C1—C6—C5	117.99 (12)	C18—C20—H20B	109.5
C1—C6—C7	117.68 (12)	H20A—C20—H20B	109.5
C5—C6—C7	124.31 (12)	C18—C20—H20C	109.5
O2—C7—C8	120.86 (12)	H20A—C20—H20C	109.5
O2—C7—C6	122.90 (12)	H20B—C20—H20C	109.5
C8—C7—C6	116.22 (12)	C22—C21—C10	112.46 (11)
C13—C8—C9	116.79 (12)	C22—C21—H21A	109.1
C13—C8—C7	121.70 (12)	C10—C21—H21A	109.1
C9—C8—C7	121.46 (12)	C22—C21—H21B	109.1
O3—C9—C10	118.70 (11)	C10—C21—H21B	109.1
O3—C9—C8	119.55 (12)	H21A—C21—H21B	107.8
C10—C9—C8	121.75 (12)	C23—C22—C21	127.38 (13)
C9—C10—C11	117.97 (12)	C23—C22—H22A	116.3
C9—C10—C21	119.71 (12)	C21—C22—H22A	116.3
C11—C10—C21	122.30 (12)	C22—C23—C24	124.58 (13)
O4—C11—C12	122.63 (12)	C22—C23—C25	121.07 (13)
O4—C11—C10	115.05 (11)	C24—C23—C25	114.35 (13)
C12—C11—C10	122.33 (12)	C23—C24—H24A	109.5
C13—C12—C11	117.62 (12)	C23—C24—H24B	109.5
C13—C12—H12A	121.2	H24A—C24—H24B	109.5
C11—C12—H12A	121.2	C23—C24—H24C	109.5
O1—C13—C12	115.91 (12)	H24A—C24—H24C	109.5
O1—C13—C8	120.60 (11)	H24B—C24—H24C	109.5
C12—C13—C8	123.49 (12)	C23—C25—H25A	109.5
O5—C14—H14A	109.5	C23—C25—H25B	109.5
O5—C14—H14B	109.5	H25A—C25—H25B	109.5
H14A—C14—H14B	109.5	C23—C25—H25C	109.5
O5—C14—H14C	109.5	H25A—C25—H25C	109.5
H14A—C14—H14C	109.5	H25B—C25—H25C	109.5
H14B—C14—H14C	109.5	O4—C26—H26A	109.5
O6—C15—H15A	109.5	O4—C26—H26B	109.5
O6—C15—H15B	109.5	H26A—C26—H26B	109.5
H15A—C15—H15B	109.5	O4—C26—H26C	109.5
O6—C15—H15C	109.5	H26A—C26—H26C	109.5
H15A—C15—H15C	109.5	H26B—C26—H26C	109.5
			
C13—O1—C1—C2	179.83 (10)	C13—C8—C9—O3	−178.46 (11)
C13—O1—C1—C6	0.85 (18)	C7—C8—C9—O3	4.11 (19)
O1—C1—C2—C3	−178.61 (11)	C13—C8—C9—C10	2.27 (18)
C6—C1—C2—C3	0.38 (19)	C7—C8—C9—C10	−175.15 (11)
C14—O5—C3—C2	3.20 (19)	O3—C9—C10—C11	−179.44 (11)
C14—O5—C3—C4	−177.28 (12)	C8—C9—C10—C11	−0.17 (19)
C1—C2—C3—O5	179.18 (11)	O3—C9—C10—C21	−0.90 (18)
C1—C2—C3—C4	−0.32 (19)	C8—C9—C10—C21	178.37 (11)
C15—O6—C4—C5	−106.85 (14)	C26—O4—C11—C12	4.87 (18)
C15—O6—C4—C3	75.47 (15)	C26—O4—C11—C10	−175.07 (11)
O5—C3—C4—O6	−1.90 (17)	C9—C10—C11—O4	177.89 (11)
C2—C3—C4—O6	177.63 (11)	C21—C10—C11—O4	−0.61 (18)
O5—C3—C4—C5	−179.54 (11)	C9—C10—C11—C12	−2.05 (19)
C2—C3—C4—C5	0.00 (19)	C21—C10—C11—C12	179.45 (12)
O6—C4—C5—C6	−177.34 (10)	O4—C11—C12—C13	−177.94 (11)
C3—C4—C5—C6	0.27 (19)	C10—C11—C12—C13	2.0 (2)
O6—C4—C5—C16	3.73 (18)	C1—O1—C13—C12	−176.43 (10)
C3—C4—C5—C16	−178.66 (11)	C1—O1—C13—C8	2.54 (17)
O1—C1—C6—C5	178.77 (11)	C11—C12—C13—O1	179.26 (11)
C2—C1—C6—C5	−0.12 (19)	C11—C12—C13—C8	0.32 (19)
O1—C1—C6—C7	−2.82 (19)	C9—C8—C13—O1	178.73 (11)
C2—C1—C6—C7	178.29 (11)	C7—C8—C13—O1	−3.85 (18)
C4—C5—C6—C1	−0.21 (18)	C9—C8—C13—C12	−2.37 (19)
C16—C5—C6—C1	178.67 (11)	C7—C8—C13—C12	175.05 (12)
C4—C5—C6—C7	−178.50 (12)	C4—C5—C16—C17	−101.46 (14)
C16—C5—C6—C7	0.4 (2)	C6—C5—C16—C17	79.66 (15)
C1—C6—C7—O2	−179.70 (12)	C5—C16—C17—C18	125.59 (16)
C5—C6—C7—O2	−1.4 (2)	C16—C17—C18—C19	−1.4 (3)
C1—C6—C7—C8	1.44 (17)	C16—C17—C18—C20	177.62 (15)
C5—C6—C7—C8	179.74 (11)	C9—C10—C21—C22	−78.58 (16)
O2—C7—C8—C13	−177.13 (12)	C11—C10—C21—C22	99.90 (14)
C6—C7—C8—C13	1.75 (18)	C10—C21—C22—C23	120.37 (15)
O2—C7—C8—C9	0.17 (19)	C21—C22—C23—C24	−0.3 (2)
C6—C7—C8—C9	179.05 (11)	C21—C22—C23—C25	−179.48 (13)

### 1-Hydroxy-3,6,7-trimethoxy-2,8-bis(3-methylbut-2-en-1-yl)-9H-xanthen-9-one Hydrogen-bond geometry (Å, º)

*Cg*1 is the centroid of the O1/C1/C6–C8/C13 ring.

**Table:**

*D*—H···*A*	*D*—H	H···*A*	*D*···*A*	*D*—H···*A*
O3—H1*O*3···O2	0.86 (2)	1.75 (2)	2.5518 (14)	155 (2)
C26—H26*B*···O6^i^	0.96	2.64	3.509 (2)	151
C15—H15*A*···O4^ii^	0.96	2.66	3.554 (2)	156
C21—H21*A*···*Cg*1^iii^	0.97	2.84	3.6949 (15)	148

Symmetry codes: (i) −*x*, −*y*+1, −*z*+1; (ii) *x*−1, *y*, *z*−1; (iii) −*x*+1, −*y*+1, −*z*+1.
